# Preservation versus Structural Cranioplasty: A New Concept for Craniosynostosis Treatment

**DOI:** 10.1055/a-2699-7853

**Published:** 2026-01-30

**Authors:** Chaline M. Matushita, Ho Hyung Lee, Jong-Woo Choi

**Affiliations:** 1 Department of Plastic Surgery, Faculdade de Medicina do ABC, Santo Andre, São Paulo, Brazil; 2 Department of Plastic and Reconstructive Surgery, University of Ulsan, College of Medicine, Seoul Asan Medical Center, Seoul, Korea

**Keywords:** preservation cranioplasty, structural cranioplasty, one-piece cranioplasty, fronto-orbital advancement, coronal, craniosynostosis, cranioplasty, distraction, bandeau

## Abstract

Craniosynostosis, a condition characterized by the premature closure of one or more cranial sutures, can be classified based on the affected suture, the number of sutures involved, or the presence of syndromic features. Although numerous surgical techniques have evolved to address cranial deformities, there remains a need for systematic categorization of these approaches. This review aims to explore current craniosynostosis treatments, evaluate their respective advantages and disadvantages, and propose a new classification distinguishing preservation cranioplasty from structural cranioplasty. The choice between preservation and structural cranioplasty is influenced by factors such as patient age, complexity of suture involvement, and resource availability. Preservation cranioplasty techniques, including suturectomy and distraction osteogenesis, prioritize maintaining cranial vault integrity and minimizing dural detachment, thus reducing surgical risks and facilitating natural cranial growth. Conversely, structural cranioplasty involves extensive bone displacement and dural exposure, offering effective correction for complex deformities and syndromic cases but with increased procedural risks. We propose categorizing craniosynostosis surgical treatments into two primary approaches: Preservation and structural cranioplasty. Preservation cranioplasty focuses on minimal invasion to support natural brain and skull development, while structural cranioplasty involves extensive remodeling necessary for severe or syndromic cases. Understanding these paradigms enables better-tailored treatment strategies and may improve long-term craniofacial outcomes.

## Introduction

The management of craniosynostosis has been the main topic of craniofacial surgery. Since the diverse forms of craniosynostosis exist, the various surgical methods and techniques have been introduced for a long time, from the simple suturectomy to total calvarial vault remodeling. However, since the numerous suggestions were made in terms of not only the surgical techniques, but also the surgical timing, we felt the need to categorize the various surgical strategies to make it clear. The review article will address and try to conceptualize the contemporary management of craniosynostosis.

In recent days, the concept and technique of preservation rhinoplasty has become very popular in contrast to the traditional structural rhinoplasty. To our understanding, preservation rhinoplasty is based on the concept of conserving as the anatomic structures as possible, while structural rhinoplasty depends on the structural graft. Likewise, we thought the preservation concept could be applicable to cranioplasty as well. Considering that most craniosynostosis correction should be done on growing children at very early ages, the preservation of the original anatomic structures would be very meaningful in the long-term craniofacial growth. In that sense, we suggest the term “preservation cranioplasty.” It includes the suturectomy, suturectomy followed by helmet molding therapy, spring-assisted cranioplasty, and distraction-based cranioplasty on the anterior and posterior cranial vault. By contrast, structural cranioplasty refers to the traditional total cranial vault remodeling and fronto-orbital advancement (FOA) with or without bandeau.

Personally, we have been actively applying not only the distraction-based cranioplasty but also the suturectomy with or without helmet molding therapy for the past 20 years. Based on the literature review, including our clinical experiences, this article will try to conceptualize the trends of modern cranioplasty.

A review of current surgical techniques in craniosynostosis is provided in Table 1 .

**Table 1:** Review of current surgical techniques in craniosynostosis

A. Preservation cranioplasty	B. Structural cranioplasty
Single suturectomy ● Suturectomy + helmet molding therapy ● Spring-assisted cranioplasty ● One-piece distraction cranioplasty	Cranial vault remodeling ● Fronto-orbital advancement with bandeau ● Posterior cranial vault remodeling ● Conventional distraction osteogenesis-based cranioplasty

Preservation cranioplasty refers to techniques that minimize surgical invasiveness—restricting osteotomy length and number, avoiding wide dural exposure, and preserving native cranial architecture. Correction relies on physiological growth vectors (brain expansion) and/or controlled external or elastic forces (helmet molding, springs, gradual distraction along intact attachments) rather than wholesale dismantling of the cranial vault.

Structural cranioplasty encompasses procedures that employ deliberate, extensive osteotomies with broad mobilization of cranial segments, detachment from the dura, and immediate reconfiguration or staged distraction of bone segments to reconstruct the desired cranial form. Stability is obtained by rigid fixation or mechanical distractors rather than primarily by intrinsic growth.

## Preservation Cranioplasty

### Suturectomy

**Fig. 1:**
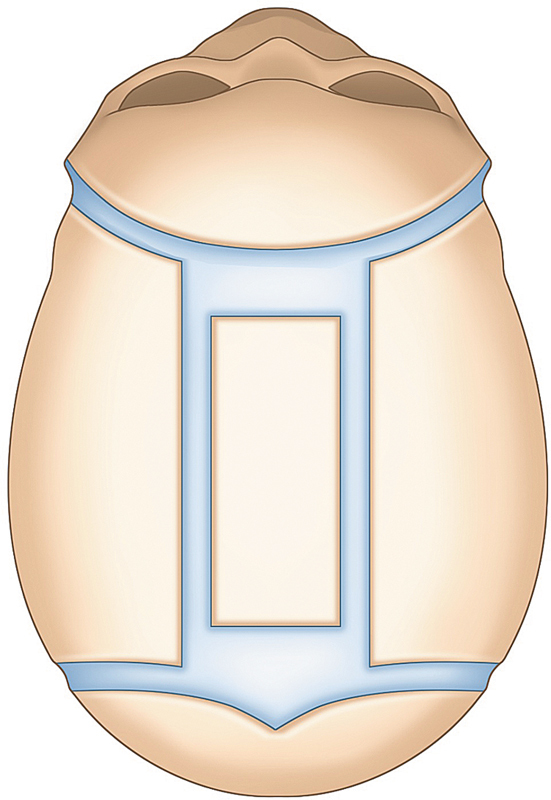
Area of suturectomy of a sagittal suture. This illustration highlights the region of the sagittal suture where an H-shaped suturectomy is performed, demonstrating the primary surgical site for treating sagittal craniosynostosis.

The simple strip suturectomy to treat craniosynostosis was first described in 1892 by Lane, 1 and by that time, his treatment was seen as a novel surgical technique to treat a cranial deformity that did not seem to have a treatment. After this first attempt, many other surgeons from all over the world reproduced his technique and some issues were found. They observed that outcomes were inconsistent and the refusion of the sutures lines occurred before the total cranial growth, limiting the final result, and recurrence of the cranial deformity was not the exception, and also, high mortality and morbidity related to this surgery. 2 Since then, this technique needed to undergo some modifications, and it was seen that for better results, the surgery had to be performed before the brain growth, therefore, before 12 months of age and also for the recalcification of the osteotomized areas ( Fig. 1 ). Renier, in 1980, published his technique to treat sagittal craniosynostosis, with an H-shaped suturectomy with six bony flaps with great results and low rate of complications when done by experienced surgeons. 3 Many advantages have been reported, such as (1) it does not require the use of plates, screws, or postoperative helmets, (2) it is proven to be a safe procedure, and (3) it has a high level of parents' satisfaction. 4 However, blood loss with the need for blood transfusion that is not suitable for children older than 12 months may be a discouraged technique for some surgeons. Some of the complications described are subgaleal hematoma, dura lacerations, and sagittal sinus lacerations ( Fig. 1 ).

### Suturectomy with “Helmet Molding Therapy”

**Fig. 2:**
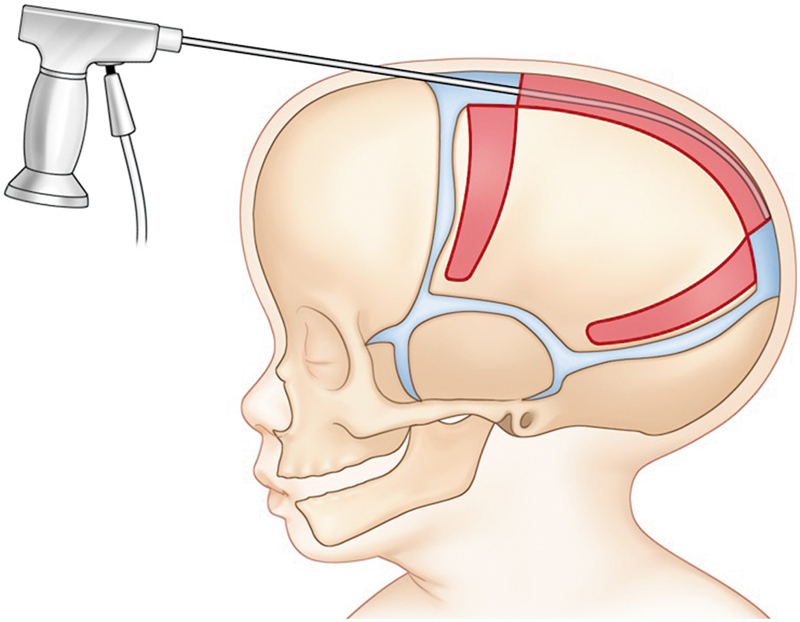
Endoscopic suturectomy. A depiction of the minimally invasive endoscopic sagittal suturectomy procedure, showing the access points used to correct craniosynostosis.

**Fig. 3:**
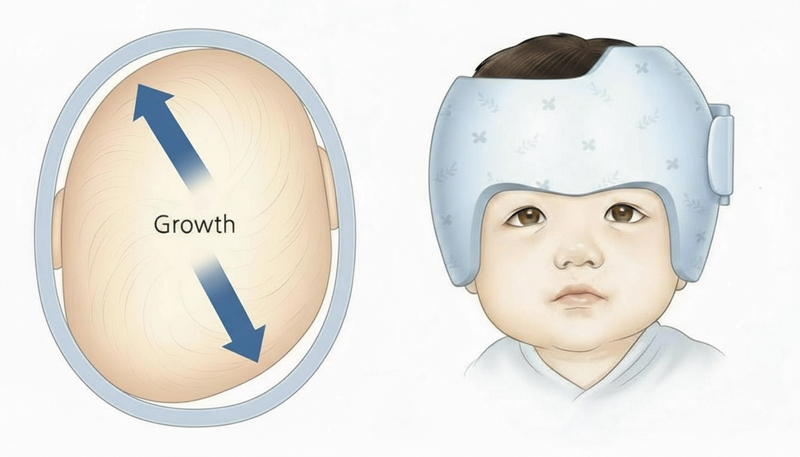
Model of a helmet molding orthosis. An illustration of postoperative helmet molding therapy used to guide skull growth and shape after suturectomy in young infants.

A minimally invasive strip craniectomy was popularized by Jimenez and Barone in 1998 when they published a four-series case of endoscopically assisted strip craniotomies to treat sagittal suture synostosis with postoperative custom cranial molding helmets. 5 Later in 2002, these same authors published a series of 100 patients treated with endoscopic strip craniectomy with orthotic molding therapy, proving the safety and advantages of this minimally invasive surgery. 6 It is performed in younger children, around 3 to 6 months old, based on Moss' functional matrix theory, 7 8 where the brain works as an internal distractor, combined with an external force applied in three dimensions by the helmet orthosis according to the children's cranial development. After that age, the cosmetic outcomes deteriorate, leading to inadequate correction of the skull shape ( Fig. 2 ). Usually, the helmet therapy will start between 10 and 14 days after the surgery and will be maintained until normocephaly has been reached or until 2 years of age, when the fastest increase in cranial volume decreases. In most institutions around the globe, the protocol is to maintain the helmet therapy for a period of 8 to 12 months. 9 10 Surgeons who advocate for the endoscopic approach with helmet therapy understand that the reducement of blood loss, hospital stay, and cost of the treatment are the major advantages of the technique; however, it is crucial to engage parents in discussions regarding their essential collaboration during the postoperative period ( Fig. 3 ). This is particularly important as several follow-up visits for orthotic treatment will be required, and the final outcomes may take time to materialize. Also, some skin alterations secondary to the helmet usage might occur, such as eczema, dermatitis, and dryness ( Figs. 2 and 3 ).

### Spring-Assisted Cranioplasty

**Fig. 4:**
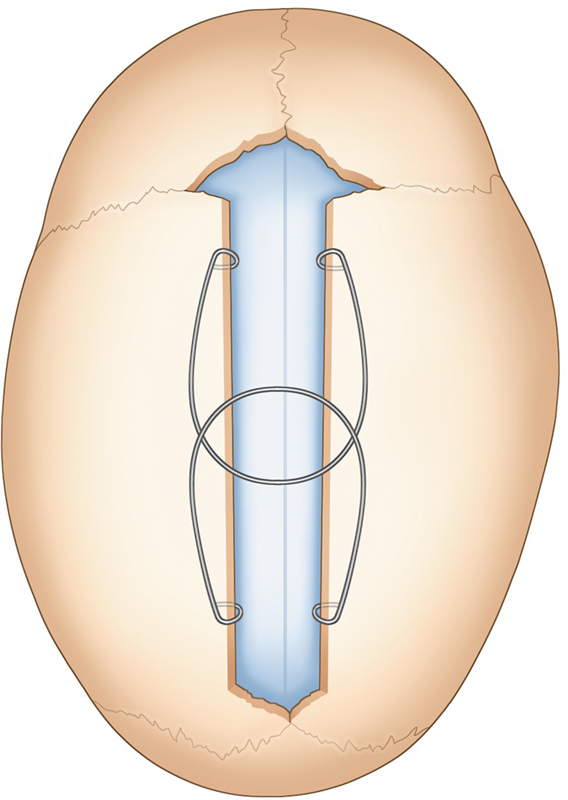
Two springs placed in the sagittal suture after suturectomy. A schematic of spring-assisted cranioplasty, demonstrating the placement of springs along the sagittal suture to facilitate controlled cranial expansion.

The use of springs was pioneered by craniofacial surgeons from Sahlgrenska University Hospital in 1997, and popularized when Lauritzen et al published, in 2008, a retrospective clinical case of 96 patients who had undergone spring-assisted cranioplasty for numerous types of craniosynostosis. 11 This technique consists of applying a spring across a strip craniectomy with little dural dissection in very young children while their cranial bone is still membranous and pliable. This spring will force both ends of the bone apart and remodel the cranial vault, serving as an opposing force between the bones against the neo-osteogenesis ( Fig. 4 ). Thereafter, a need is to remove the spring with a second surgery with general anesthesia. Spring removal occurs when reossification of the calvarial defect occurs, approximately after 3 to 4 months. 12 13 14 15 Its best indication is for the treatment of sagittal synostosis in children between the ages of 4 and 6 months, 14 15 16 17 but it can be used to treat many other different types of craniosynostosis as well as posterior vault expansion. 16

Some of the known complications found in the literature are spring dislocation, erosion of overlying skin, skin penetration, difficulties in removing the springs, irregular cranial shape, and elevation of intracranial pressure (ICP) when used as compressive transverse springs in cases such as brachycephaly. 11 12 Moreover, the necessity of a secondary surgery to remove the spring, the possibility of undercorrection, and the need for further long-term studies about this technique seem to be the main reason for some surgeons not to use this strategy. However, surgeons who perform this surgery say that the advantages; minimum dural dissection the blood loss is minimized and also the need for blood transfusion, 11 12 better team dynamics between neurosurgery and craniofacial surgeons, 18 can be performed at the age gap between the best timing indication for endoscopic approach and cranial vault reconstruction, smaller incision, and less dural dissection and the **risk** of dural **tear** and fistula, 12 outweigh the disadvantages ( Fig. 4 ).

### Distraction-Based Cranioplasty

**Fig. 5:**
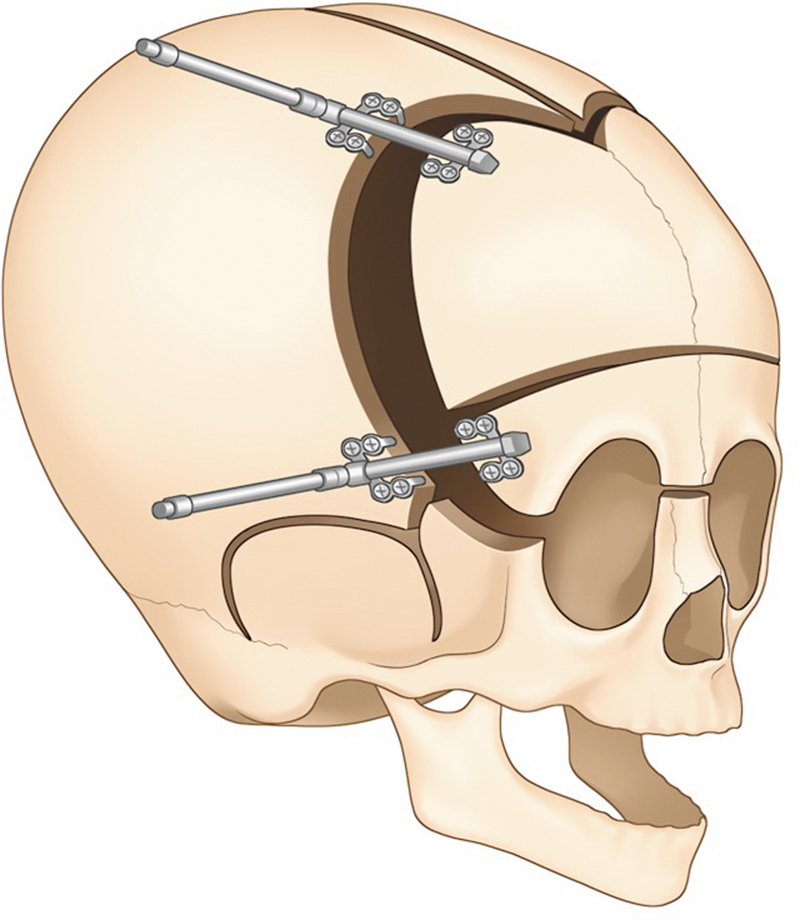
Fronto-orbital advancement with distractors. This image illustrates the use of distractors for fronto-orbital advancement, a technique that gradually moves the frontal bones forward for improved cranial reshaping.

In 2000, Matsumoto et al described the first use of distraction osteogenesis (DO) for advancement and monobloc distraction of a Pfeiffer syndrome patient. 19 Since then, numerous papers have reported on the correction of sagittal craniosynostosis. By that time, DO used for mandible and midface have shown the beneficial aspects of the use of distractors to correct the premature fusion of the cranial sutures. This technique consists of a suturectomy followed by the insertion of one or more distractors that are screwed at the two ends of the cranial bones, to drive apart as the distraction occurs after an average period of 5 to 7 days. Distraction will happen slowly at the rate of 1 to 1.5 mm/day by the care provider of the patient. Since it is a technique that does not rely on brain growth, it is possible to perform it in older children ( Fig. 5 ).

In 2009, Choi et al published a new technique to treat the unilateral coronal craniosynostosis using a one-piece FOA method. In this method, the bone flap was not separated from the dura, and with a single burr hole at the pterion, a coronal osteotomy was performed, and a small hole was made in the skull. The bone was then cut along the planned lines at the nasofrontal, orbital roof, zygomaticofrontal, and sphenofrontal sutures. Their results showed that the one-piece fronto-orbital distraction had better outcomes regarding the correction of the posterior and anterior cranial fossa compared to the traditional bone reposition techniques. 20

**Fig. 6:**
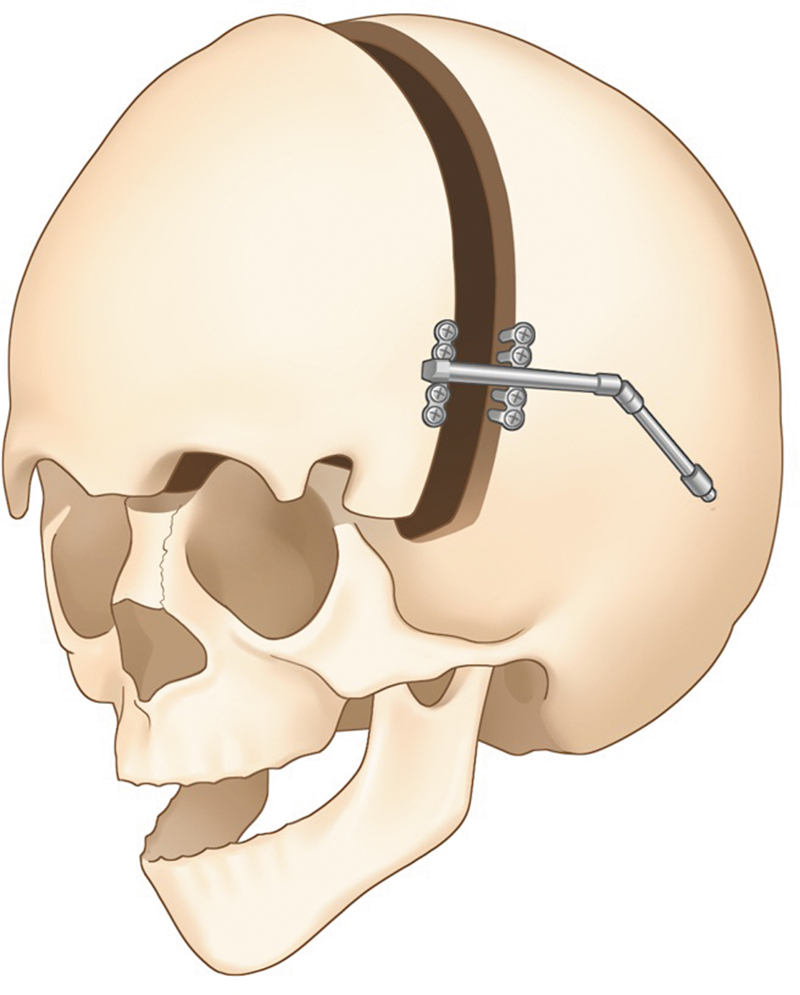
One-piece advancement technique by Choi et al (2009), 20 without a fronto-orbital bandeau. A visual representation of the one-piece fronto-orbital advancement technique, showing its impact on cranial morphology and how it differs from traditional methods.

One year later, in 2010, Choi et al evidenced in their paper the presence of a greater skull base axis change with the one-piece distraction technique, which is a form of one-piece cranioplasty, resulting in a greater correction of the plagyocephaly. 21 It has already been well-established that the advantages of the DO techniques are numerous, such as reduction of the postoperative extradural dead space, preservation of the blood supply of the bone flap from the dura, shortage of the operative time, and absence of the necessity of bone grafts. In spite of that, infections can occur near the device, a second surgery is needed to remove the distractor, a scar is produced where the device is externalized, and the treatment period is prolonged and also relies on parental compliance for the distraction ( Fig. 6 ).

White et al, in 2009, described six patients' cases who underwent posterior cranial vault DO due to raised ICP, with attractive and promising results, with a reasonable increase in intracranial volume that overcame one of the main difficulties of open remodeling, which is the limitation of the infant's soft tissue. 22 Afterward, several surgeons began to implement this new technique, and it has proved to be a validated method for enhancing intracranial volume, reducing ICP, and effectively addressing turribrachycephaly. The appropriate age for performing the surgery varies in the literature, typically ranging from 3 to 9 months. This variability depends on the severity of ICP as well as the protocols established by the institution and/or surgeon. 23 Since the calvarial bone flaps remain attached to the dura, the risk of devascularization and bone loss is theoretically reduced compared with conventional cranial vault remodeling techniques. However, complications regarding this surgery have been reported, such as postoperative infection, cerebrospinal fluid leak, and device failure. 23

During the first 2 years of life, the brain undergoes significant growth, tripling in size, with the most substantial development occurring during the first year, particularly in the posterior half of the skull. It is well-established that certain syndromes, such as Pfeiffer, Crouzon, and Apert, are associated with an increased risk of excessive intracranial enlargement. Due to the disproportionate growth of the posterior skull, posterior cranial remodeling has been identified as an effective strategy for alleviating elevated ICP. This approach not only addresses urgent concerns but also provides a more efficient method of advancing the cranial vault compared with traditional anterior remodeling.

For example, in the treatment of conditions like turricephaly and bilateral coronal craniosynostosis, posterior cranial remodeling not only helps alleviate symptoms of increased ICP but also improves cranial shape. The development of cranial osteogenic distraction specifically targeting the posterior region has significantly reduced many of the challenges previously encountered with open conventional approach. This method preserves bone vascularization, allows for gradual soft tissue expansion, and reduces tension during wound closure, which in turn minimizes the risk of dehiscence. These advantages, alongside the benefits of cranial vault DO, have further enhanced the efficacy and outcomes of cranial remodeling procedures.

The posterior cranial vault DO technique involves positioning the patient in a prone posture. A bicoronal incision is made, followed by an osteotomy extending from the biparietal vertex to the torcula. Two to three distractors are placed in the parasagittal region, ensuring that they are fixed in parallel alignment to prevent the vectors from converging. 24

## Structural Cranioplasty

### Total Vault Cranial Remodeling

Total cranial vault remodeling is a surgical technique in which the bones of the cranial vault are detached from the dura mater and, through a series of osteotomies, are reshaped to expand the cranial vault and achieve a satisfactory aesthetic outcome. A variety of surgical approaches have been described for treating different types of craniosynostosis. Specifically, in the management of scaphocephaly, multiple osteotomy designs have been proposed. However, there is no consensus on a universally superior technique, and the choice of the most appropriate approach ultimately rests with the surgeon. Several techniques have been outlined in the literature for addressing scaphocephaly, including the pi procedure with or without barrel stave osteotomy, parietal reshaping with barrel stave osteotomy, geometric parietal expansion with barrel stave osteotomy, parietal–occipital switch with barrel stave osteotomy, clamshell craniotomy with barrel stave osteotomy, and strip sagittal suturectomy with barrel stave osteotomy. These examples represent some of the surgical options available for the treatment of scaphocephaly. 25

The Melbourne technique, introduced in 2008, was designed to offer improved correction for severe cases of scaphocephaly or those that present later in development. This technique involves a novel approach in which a precoronal frontal segment is detached, combined with barrel staving to address **frontal bossing** and supraorbital bar burring for contouring. For lateral and occipital deformities, the procedure includes en bloc removal and elevation of the occiput, along with the advancement and repositioning of osteotomized bones, which are then fixed with plates.

**Fig. 7:**
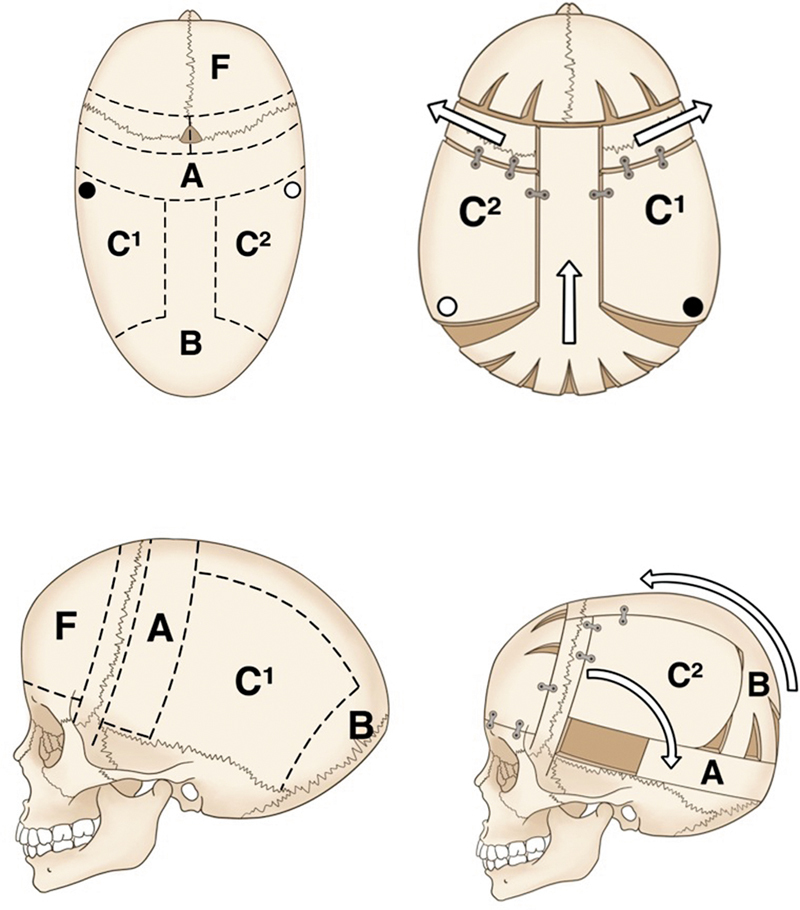
The Melbourne method of total vault remodeling for severe scaphocephaly. Inspired by Greensmith et al, 26 this illustration outlines the steps of total vault remodeling for correcting severe cases of scaphocephaly, including lateral and occipital deformity adjustments.

A key advantage of this technique is that it is claimed to effectively treat all phenotypes of scaphocephaly, achieving good results with minimal complications. However, the original study lacks long-term follow-up data and a larger patient sample, which are necessary for a more comprehensive understanding of the long-term outcomes 26 ( Fig. 7 ).

The most complex cases of craniosynostosis are often associated with syndromes such as Crouzon, Pfeiffer, Apert, Muenke, and Saethre–Chotzen, which are the five primary syndromes characterized by multisuture craniosynostosis. In these cases, the involvement of multiple sutures may necessitate more than one surgical intervention. The initial surgeries are typically performed with greater urgency due to the risk of increased ICP, which can lead to serious complications, including psychomotor developmental delays and visual impairment from papilledema. These urgent procedures are followed by later interventions, such as cranial remodeling and monobloc frontofacial advancement, typically performed from the age of 5 years onward. The monobloc advancement is often combined with frontoplasty and is specifically aimed at correcting hypoplasia of the middle third of the face, improving respiratory function, and further enhancing facial aesthetics and structure. 27

### Fronto-orbital Advancement with Bandeau

The FOA is mostly performed to correct unicoronal, bicoronal, and metopic craniosynostosis with deformity of the forehead and superolateral orbit. It is a technique with a considerable detachment of the scalp from the cranial bone, from the posterior vertex to the frontonasal suture, and from the zygomaticofrontal suture to the squamosal suture. It consists of bilateral frontal craniotomy for suture release and decompression, combined with the creation of a “supraorbital bar” as a bilateral orbital complex by osteotomizing the orbital roof (anterior cranial base), supraorbital ridge, and upper lateral orbital rim bilaterally. 28 29 30 31 This is followed by a bilateral advancement and remodeling of the frontal region as well as the orbital region bilaterally, which is then rigidly fixed in position, the supraorbital bar to the face (at the fronto-zygomatic region and the fronto-nasal region), and the reconstructed forehead to the supraorbital bar.

**Fig. 8:**
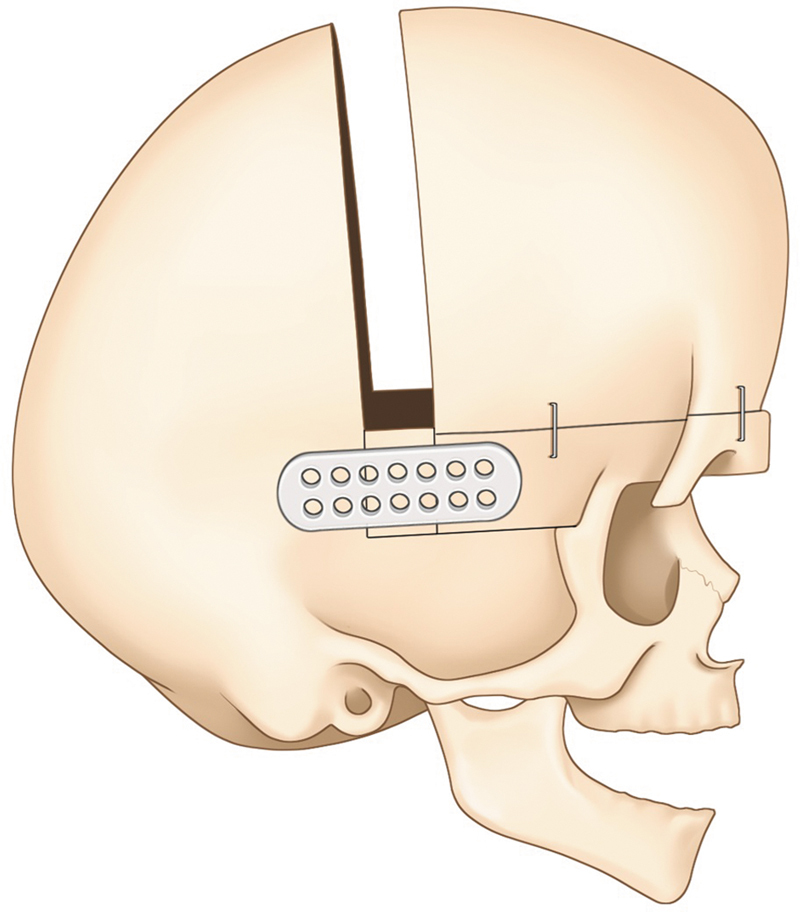
Conventional open fronto-orbital advancement with plates for stabilization. This image depicts the traditional fronto-orbital advancement technique, showing the placement of fixation plates and the extent of dura dissection.

For this extensive cranial detachment, interventions are usually postponed until 6 months. It is generally understood that at this age, children are more capable of handling major surgeries, reducing the risk of increased pressure in the brain, ensuring proper support of the frontal–orbital bar, benefiting from the natural growth and changes in their brains, and attaining more favorable cosmetic results. 32 Its versatility for the treatment of all degrees of cranial deformity and especially for syndromic cases, makes this technique one of the most performed since it is well-known, predictable, and gives good results. Nonetheless, the disadvantages cannot be underestimated, including blood loss transfusion, longer hospital stay, prolonged surgery duration, and older age of treatment that can cause parental anxiety ( Fig. 8 ).

### Open Posterior Cranial Vault Remodeling

This technique can be divided into a one-staged procedure for the treatment of uni- or bilateral lambdoid craniosynostosis or as a two-staged surgery for patients with increased ICP. As a one-staged surgery, many techniques have been described where the occipital and parietal bone are removed en bloc and then divided, remodeled, and shaped ex vivo. The affected side will receive the non-affected side of the bone, whereas the non-affected side will receive the contralateral side. This switch on the bones' sides will provide volume to the previously troubled side. Also, it is common to see a **parietal bossing** at the contralateral side, so barrel stave osteotomies might be necessary for a favorable outcome. It is mandatory to be extremely cautious with the torcula and avoid its injury. As a two-staged surgery for patients with elevated ICP secondary to various types of craniosynostosis, especially in syndromic patients, multisuture craniosynostosis and rare synostosis such as the Mercedes-Benz pattern, also known as “posterior trigonocephaly” and the “z” pattern craniosynostosis. The main goal of this first step is to manage the ICP to favor psychomotor development and also ocular protection.

**Fig. 9:**
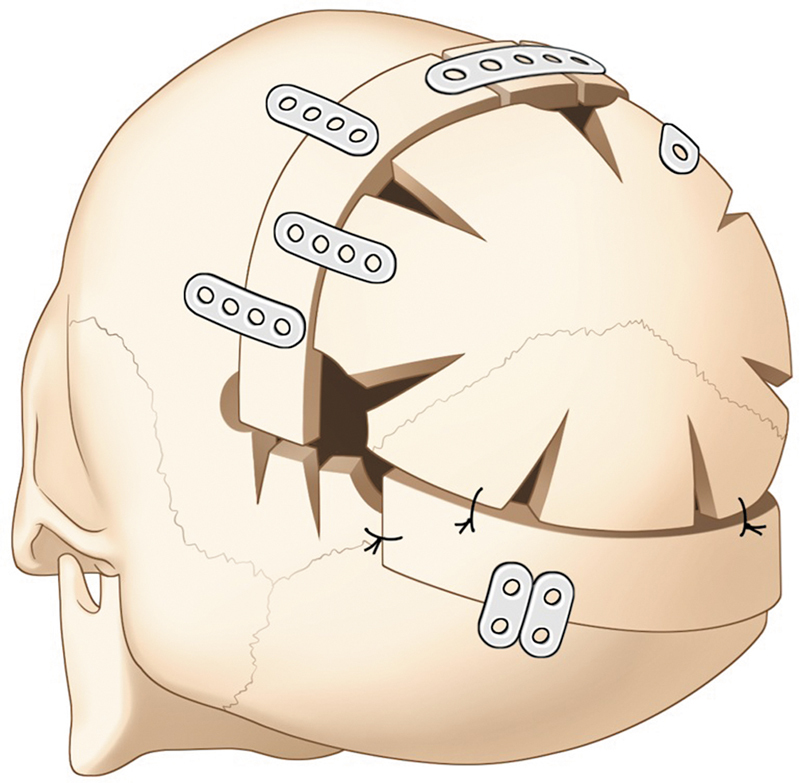
Open posterior cranial vault remodeling. A schematic of the open posterior cranial vault remodeling procedure, detailing the osteotomies and bone movements used to expand the cranial vault.

Complications regarding the structural cranioplasty approach can occur early or late. Bleeding, cerebral fluid leaking, dura mater tearing, infection, meningitis, and air embolism to the dural sinus are acute complications with low prevalence and a mortality rate below 1%, 33 yet late complications would be the absence of reossification and irregularities in head shape, and both can lead to reoperation ( Fig. 9 ).

## Discussion

Numerous studies demonstrate the differences and effectiveness of various techniques in comparison to one another. Many of the most renowned studies supporting the concept that minimally invasive surgeries are more advantageous for patients—due to reduced blood loss, shorter hospital stays, and lower health care costs—are conducted within tertiary care facilities that often possess financial resources not universally available. However, it is imperative to consider additional factors when evaluating the optimal treatment for patients.

A critical factor in determining the appropriate treatment for patients is the age at which the diagnosis is made and the timing of the consultation with a neurosurgeon or craniofacial surgeon. Unfortunately, it is not always the case that these patients receive a diagnosis during the early months of life. This delay may arise from pediatricians or general practitioners—who are typically the first point of contact during routine consultations—failing to recognize the condition, or from a delay in obtaining the appropriate referral to a specialist capable of addressing and managing craniosynostosis. Delayed presentation may be associated with a higher occurrence of intracranial hypertension, which can manifest as headaches, changes in personality, and/or mental decline. 34 An interesting study conducted in India, published by Seruya et al, with 211 patients, showed that the mean age of initial consultation for sagittal craniosynostosis was **3.2 months** , metopic **4.4 months** , and even though the gross cranial deformity, multiple sutures synostosis had their consultation at **10.7 months** . 34

Late diagnosis has a direct impact on the selection of available treatment options, often precluding the possibility of endoscopic surgery with helmet therapy. This surgical approach is associated with more favorable outcomes when performed on children, ideally by the age of 3 months, although the literature suggests that it may still be effective when conducted up to 6 months of age. It is anticipated that advancements in technology and the education of health care professionals will lead to an increase in early diagnosis of craniosynostosis. Prenatal diagnosis via ultrasound can serve as a valuable tool for parents, 35 enabling them to arrange a consultation with a specialist in the initial months of the child's life. This proactive approach allows for the clarification and discussion of all types of surgical options between the surgeon and the parents.

Another significant consideration is the challenge that many surgeons face in obtaining authorization from health insurance plans to use specific treatment techniques. For instance, the employment of distractors, springs, and helmet therapy is often restricted, despite the surgeon's clinical preference and the relevant indications. In many countries, agreements may prohibit the use of these devices due to their high cost, even though the overall length of hospital stay and the required medical materials may be reduced as a result. A study conducted in Alabama (the United States), which is regarded as a developed nation, revealed that the Black population and individuals reliant on Medicaid in that region face significant barriers to accessing less invasive procedures, such as endoscopic surgery. This difficulty arises primarily from delays in obtaining insurance authorization, which prevents timely surgical intervention. Additionally, the lack of coverage for necessary helmets, critical for the success of such surgeries, further exacerbates these challenges. The findings of this study were so impactful that they led to changes in Medicare policies in Alabama. 36

Numerous cases of craniosynostosis present distinct challenges due to their complexity, often resulting in prolonged surgical durations and significant blood loss, particularly for pediatric patients who are generally too young to endure such intricate procedures. However, advancements in technology have facilitated the increasing adoption of virtual surgical planning, aimed at minimizing both surgical and hospitalization length for patients undergoing structured cranioplasty. This innovative approach enables the utilization of intraoperative guides for osteotomies, tailored to achieve the desired outcomes proposed in preoperative planning, thereby enhancing precision in surgical results. Furthermore, meticulous planning serves as a valuable tool for anticipating overcorrection in light of the child's growth.

Despite acknowledging the limitations inherent to each institution and country, our institution has adopted the concepts of preservation and structural cranioplasty. Whenever feasible, we prioritize preservation cranioplasty, aiming to minimize disruption to healthy tissue and reduce bone devascularization, thereby mitigating complications commonly associated with structured cranioplasties. Nonetheless, we also recognize the critical role of structural cranioplasty, particularly in secondary cases and those involving syndromic conditions, **frontal bossing** , or contouring deformity.
